# High Drying Temperature Accelerates Sunflower Seed Deterioration by Regulating the Fatty Acid Metabolism, Glycometabolism, and Abscisic Acid/Gibberellin Balance

**DOI:** 10.3389/fpls.2021.628251

**Published:** 2021-05-28

**Authors:** Yutao Huang, Min Lu, Huaping Wu, Tiyuan Zhao, Pin Wu, Dongdong Cao

**Affiliations:** ^1^Institute of Crop and Nuclear Technology Utilization, Zhejiang Academy of Agricultural Sciences, Hangzhou, China; ^2^Huzhou Keao Seed Co., Ltd., Huzhou, China

**Keywords:** drying temperature, fatty acid, phytohormones, ROS, seed germination, seed storage, sunflower

## Abstract

Sunflower seed storage is accompanied by the loss of seed vigor. Seed drying is a key link between seed harvest and seed storage; however, to date, the effect of seed drying on sunflower seed deterioration during storage remains unclear. The present study performed hot air drying for sunflower seeds with an initial moisture content of 30% to examine the manner in which drying temperature (35, 40, 45, 50, and 55°C) affects the drying performance and seed vigor following storage process (6 and 12 months). A drying temperature of 40°C was evidently safe for sunflower seeds, whereas the high drying temperatures (HTD, 45, 50, and 55°C) significantly lowered sunflower seed vigor by regulating the fatty acid metabolism, glycometabolism, and abscisic acid (ABA)/gibberellin (GA) balance. HDT significantly increased the seed damage rate and accelerated sunflower seed deterioration during natural and artificial aging process. Further biochemical analysis indicated that HDT significantly increased lipoxygenase and dioxygenase activities, leading to malonaldehyde and reactive oxygen species over-accumulation during storage. During early seed germination, HDT significantly inhibited fatty acid hydrolysis and glycometabolism by decreasing triacylglycerol lipase, CoA-SH oxidase, and invertase activities. Moreover, HDT remarkably increased ABA levels but reduced GA levels by regulating gene expressions and metabolic enzyme activities during early imbibitions. Cumulatively, the seed drying effect on sunflower seed vigor deterioration during the storage process may be strongly related to fatty acid oxidation and hydrolysis metabolism, toxic substance accumulation, and ABA/GA balance.

## Introduction

Sunflower (*Helianthus annuus* L.), originally native to North America, represents an important oilseed crop in the world (Buti et al., 2011). Further, sunflower is the third most important oil crop in China, with over 1.15 million hm^2^ planting area and 299 t overall yield (Zhang and Zhang, 2018). Sunflower has recently been gaining substantial attention because of its highly edible oil production and ornamental values.

Seed vigor is indicated by more rapid germination or greater tolerance of stressful conditions (Rajjou et al., 2012). Furthermore, seed longevity plays a crucial role in seed vigor, determined by seed physiological, genetic characteristics and storage conditions (Dang et al., 2014). High seed vigor is strongly related with excellent field emergence and productivity, whereas low seed vigor results in decreased output. Seed moisture can affect seed vigor in stored seeds; thus, it should be kept in an appropriate range for seed storage (Akpinar, 2005; Bie et al., 2007). Normally, sunflower seed are usually harvested with lower initial content (<25%) in main sunflower producing areas in northern China. However, harvested sunflower seeds have a high initial moisture content (IMC) ranging from 20 to 35% in southern China, especially in Zhejiang province, mainly due to the influence of the rainy season. A high IMC may result in microbial proliferation, thereby causing deteriorated seed vigor. Ideally, a moisture content of 9.0% is considered appropriate for sunflower seeds (Zhen et al., 2020). Therefore, it is important to conduct research on seed drying for the reproduction of high-vigor sunflower seeds.

Seed drying involves a non-linear process as it is delayed for a long term and is extremely complicated. IMC, drying temperature and drying environment are the key parameters that affect seed response to hot air drying. Drying temperature regulated seed vigor, and regulates the seed temperature and drying speed. Drying at high temperatures may lead to seed stress cracks, enzyme activity dysregulation, and seed vigor loss (Igathinathane et al., 2008). Moreover, it may decrease seed vigor *via* suppressing storage substance catabolism in the seed endosperm. Besides, the impact of drying temperature on seed germination depends on the IMC, the higher the IMC the higher the effect (Huang et al., 2020b). Seeds with high IMC are likely to cause seed vigor degradation with a high drying temperature (Thakur and Gupta, 2006). A lower drying temperature should be applied for high IMC seeds to ensure seed vigor (Zhou et al., 2018).

Seed vigor deterioration routinely occurs during the seed storage process, closely associated with seed property, storage conditions, and storage duration (Faligowska et al., 2012; Zhao et al., 2017). However, there is no report on the mechanism *via* which drying temperature affects seed vigor deterioration during the crop seed storage process, particularly in oilseeds. Oilseeds are rich in unsaturated fatty acid, and the oxidation of unsaturated fatty acids can easily cause cell membrane permeability. Lipoxygenases (LOXs) play a vital role in lipid peroxidation of the seeds of rice (*Oryza sativa* L.) (Xu et al., 2015), soybean (*Glycine max* L.) (Lima et al., 2010), sweet lupin (*Lupinus* L.) (Stephany et al., 2015), and canola (*Brassica napus* L.) (Terp et al., 2006). LOX catalyzes oxygenation of polyunsaturated fatty acids (PUFAs), including linoleic and linolenic acids, to form conjugated diene hydroperoxides. Furthermore, lipid oxidation produces peroxides and free radicals, which cause damage to DNA, proteins, membrane structures, and cell tissues, thereby inducing seed vigor loss (Chen et al., 2010). Zhou et al. (2019) reported that the processes of triacylglycerol and fatty acid hydrolysis were blocked during the early germination stage of the aging soybean seeds, and it was the primary cause for the reduced soybean seed vigor following storage.

Seeds contain a large amount of macromolecules nutrients such as fat, starch, and protein. Such macromolecules can be gradually decomposed and used in germination (Xiong et al., 2019). Triacylglycerol is the primary storage lipid in oilseeds, and its decomposition plays a vital role in oilseed germination, including sunflower and soybean seed. In early germination process, triacylglycerol undergoes direct hydrolysis to form glycerin and fatty acids *via* lipase. Fatty acids generate CoA-SH *via* theβ-oxidation pathway, which further produces oxaloacetic acid *via* the glyoxylate or tricarboxylic acid cycle and is finally reversely converted to sucrose *via* glycolysis (Goepfert and Poirier, 2007). Glycerin is another triacylglycerol hydrolysis product that can produce phosphoglyceride catalyzed byglycerol-3-P dehydrogenase (GPDH) and subsequently generate dihydroxyacetone phosphate (DHAP) *via* dehydrogenation. DHAP is eventually converted to glucose and used in seed germination (Quettier et al., 2008).

Gibberellin (GA) and abscisic acid (ABA) are two vital phytohormones involved in the seed dormancy and germination. GA is responsible for seed dormancy breaking and seed germination induction and the subsequent activation of seed endogenous hydrolase activity that results in the promotion of storage substance cleavage (Rajjou et al., 2012). GA biosynthesis in higher plants is primarily catalyzed by GA20-oxidase (GA20ox) and GA3-oxidase (GA3ox), whereas GA catabolism is catalyzed by GA2-oxidase (GA2ox) (Hedden and Thomas, 2012). In comparison, ABA plays a major role in seed dormancy. ABA accumulation leads to seed dormancy, whereas its deficiency provokes non-dormancy phenotypes. Zeaxanthin epoxidase (ZEP), abscisic aldehyde oxidase (AAO), and 9-*cis*-epoxycarotenoid dioxygenase (NCED) represent three main enzymes responsible for ABA biosynthesis in higher plants, and ABA-8′-hydroxylase (ABA8ox) is responsible for ABA catabolism (Vishwakarma et al., 2017).

To date, research regarding the optimal sunflower seed drying temperature is scarce. Moreover, the regulatory mechanism of drying temperature on sunflower seeds vigor during storage and germination has not yet been thoroughly examined. In the present study, the effects of air drying temperature (35, 40, 45, 50, and 55°C) on drying performance and seed vigor of sunflower seeds during storage and early germination were investigated. In addition, the reactive oxygen species (ROS) metabolism, fatty acid metabolism, glycometabolism, and plant hormone metabolism of sunflower seeds during storage and germination was assessed. The present research may provide a future reference to design of a drying technology for sunflower seeds and enrich the regulatory mechanism underlying the effect of drying temperature on crop seed vigor.

## Materials and Methods

### Materials

Seeds of the sunflower (*Helianthus annuus* L.) cultivar “Aidatou” were used in the present study. Seeds with an IMC of 30% were harvested on October 13, 2018. The IMC was measured using a corn moisture apparatus (GAC-2100AGRI, Tuopu, Hangzhou, China), with ten biological replications. The weather conditions during harvest are shown in Supplementary Table 1. Sunflower seeds were dried with the Sanjiu low-temperature dryer (NEW PRO-120 H).

### Drying Experiment

Sunflower seeds with an IMC of 30% were dried until a moisture content of 9% was achieved at 35, 40, 45, 50, and 55°C. Seed samples were taken every 6 h during the drying process. Subsequently, the damaged seed number was calculated. Seeds with visually observable cracks were considered as damaged seeds. Each drying experiment was performed in triplicate under every drying temperature.

### Seed Storage

The dried sunflower seeds were stored in a dry, airtight container at room temperature for 1, 6, and 12 months. Seed samples were collected and used for the germination test and further determinations.

### Accelerated Aging Test

The accelerated aging test (AAT) was performed with the method of Missaoui and Hill (2015). Briefly, 100 sunflower seeds were added into a seed aging box for a 48-h incubation under 45°C and 100% relative humidity conditions (AAT conditions were decided based on preliminary experiments). Thereafter, seeds were dried at room temperature for 2 days before the germination tests. The accelerated aging test was performed with four biological replicates.

### Seed Germination Test

Seed germination test was conducted after disinfection of the seeds with 0.1% sodium hypochlorite solution for 15 min. Sunflower seeds were added into rolled towels (with 50 seeds in each towel) and subjected to 5 days of incubation within the germination chamber under 25°C and 12-h/12-h light/dark cycle conditions. The germinated seed number was calculated on days 3 and 5, and seed samples were sampled on days 1 and 3 during germination. The seed germination test was performed with four biological replicates.

### H_2_O_2_, O_2_^–^, and Malondialdehyde Analysis

H_2_O_2_ content was analyzed with the method of Huang et al. (2011) based on absorbance measurement of the supernatant-measured as its optical density (OD) at 390 nm. In addition, O_2_^–^ level was determined with the method of Jiang and Zhang (2003) based on supernatant OD at 530 nm. Malondialdehyde (MDA) level was detected according to the method reported by Gao et al. (2009) and calculated based on supernatant OD at 532 and 600 nm. The analysis of H_2_O_2_, O_2_^–^, and malondialdehyde was performed with four biological replicates.

### Quantification of Various Sugars

Anthrone-H_2_SO_4_colorimetry was applied for determining the concentration of total soluble sugar in sunflower seeds according to Zhu et al. (2015). In addition, sucrose concentration was measured using the resorcinol approach based on OD at 480 nm (Shi et al., 2016). The fructose concentration was measured using a previously reported approach (Cai et al., 2016). Glucose level was analyzed with high performance liquid chromatography (HPLC) according to Bailly et al. (2010). The quantification of various sugars was performed with four biological replicates.

### ATP and Energy Charge Analysis

High performance liquid chromatography was performed to determine ATP and energy charge according to Liu et al. (2006). Data of ATP and energy charge analysis were expressed as means of four replicate determinations. The analysis of ATP and energy charge was performed with four biological replicates.

### Fatty Acid Concentration Measurement

Sunflower seed samples were grinded to a powder form for fatty acid extraction. Briefly, 2 mL of n-hexane was added to 50 mg sunflower seed powder in each tube, followed by 15 min of ultrasonic extraction (40 kHz) and 3 h of incubation at room temperature. Thereafter, the mixed solution was subjected to 10 min of centrifugation under 4°C and 10,000 rpm. The supernatants were collected and subjected to vortex oscillation for 30 s after the addition of 3 mL methanolic potassium hydroxide solution (0.6 M). Thereafter, the supernatant was collected and added to a 5-mL bottle, and n-hexane was used to dilute the solution to a total volume of 5 mL. Subsequently, a 0.45-μm filter (organic phase) was used to add the extract into the gas chromatography–mass spectrometry (GC-MS) system. Finally, fatty acid concentration was test with the method described by Yang et al. (2017). The fatty acid concentration measurement was performed with four biological replicates.

### ABA and GA Level Determination

The ABA and GA extraction from sunflower seeds was performed using the method of Huang et al. (2017). Typically, ABA and GA concentrations within extracting solution were determined using the HPLC system using a reversed phase column (C18, 6.0 mm × 120 mm, particle size 5 mm; Shim-Pack CLC-ODS) and an ultraviolet detector. Meanwhile, the mobile phase composed of methanol/water (64:36, v/v) was adopted with a flow rate of 1 mL⋅min^–1^. ABA and GA levels were determined from four biological replicates.

### Assay of Fatty Acid Metabolism-, Glycometabolism-, and ABA and GA Metabolism-Related Enzyme Activity

The activities of lipoxygenase (LOX), dioxygenase (DOX), triacylglycerol lipase (LIPG), GPDH, CoA-SH oxidase (ACX), phosphoenolpyruvate carboxykinase (PCK), invertase (INV), NCED, ZEP, AAO, ABA8ox, GA3ox, GA20ox, and GA2ox were detected using an enzyme-linked immune assay kit (Mlbio, Shanghai, China) according to the kit introduction. Following the chromogenic reaction, an enzyme mark instrument was used to spectrophotometrically measure the color change at 450 nm. The activities of enzymes related to fatty acid metabolism, glycometabolism, and ABA and GA metabolism were determined according to the OD of the samples relative to the standard curve. The enzyme activity analysis was performed with four biological replicates.

### Gene Expression Analysis

The PrimeScript^TM^ RT Reagent Kit (Vazyme, Nanjing, China) was used for RNA extraction from seed samples and cDNA synthesis *via* reverse transcription. The CFX96 Touch Real-Time PCR machine (Bio-Rad) was adopted for quantitative polymerase chain reaction (qPCR) procedure using the ChamQ SYBR qPCR Master Mix (Vazyme). Supplementary Table 2 presents all primers used in the present study; 18srRNA was used as the endogenous reference. The fold change (FC) in expression was calculated as follows: FC = EΔCt, where E indicates the mean gene amplification efficacy and ΔCt stands for the difference in mean Ct values (obtained from all duplicates). The values were presented in the manner of mean SD after normalization. The gene expression analysis was performed with four biological replicates, and each was made in three technical replicates.

### Statistical Analyses

Data were statistically analyzed with one-way ANOVA using the Statistical Analysis System software. Further, the least significant difference of p value of < 0.05 (LSD_0.05_) was adopted for multiple comparisons. Percentage data were converted using the arcsine transformation before statistical comparison as follows: ŷ = arcsine[sqrt (x/100)].

## Results

### HDT Decreased the Moisture Content and Increased the Seed Deterioration Rate in Sunflower

**FIGURE 1:**
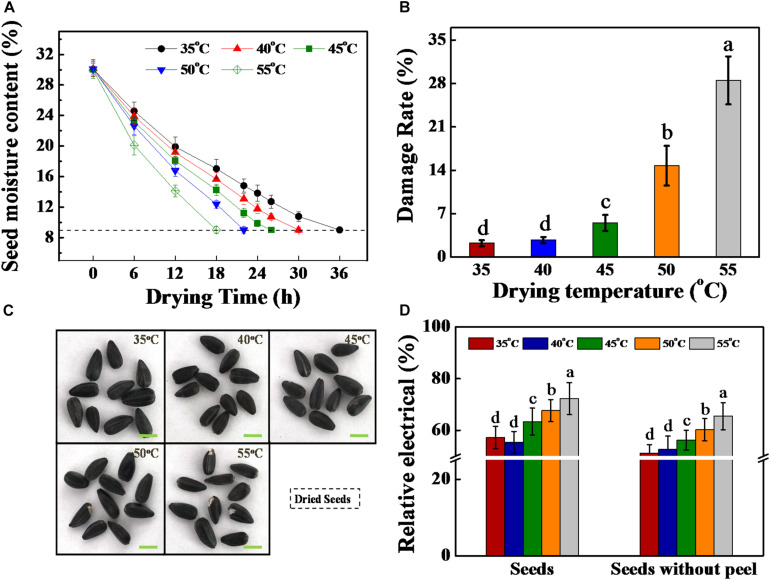
High drying temperature decreased the moisture content and increased the damage rate of sunflower seeds. **(A)** Seed moisture content during drying stage. **(B)** Damage rate of dried sunflower seeds after drying. **(C)** Photographs of dried sunflower seeds after drying. **(D)** Relative electrical of dried sunflower seeds. Sunflower seeds were dried to a target moisture content of 9% at temperatures of 35, 40, 45, 50, and 55°C. Percentages represent the means from four experiments ± SE. The diverse small letter(s) on top of the bars indicate significant differences (*p* < 0.01, LSD) between treatments. Each drying experiment was performed in three biological replicates.

The sunflower seed drying rate and equilibrium temperature showed substantial increase with the increase in drying temperature. Consequently, the drying time significantly declined with the increase of drying temperature (Table 1). The drying time required for reaching the target seed moisture content of 9% was 36.1, 30.3, 26.2, 22.0, and 18.2 h at drying temperatures of 35, 40, 45, 50, and 55°C, respectively (Figure 1A).

**TABLE 1:** High drying temperature decreased drying time and increased drying rate and seeds temperature of sunflower seeds.

**IMC (%)**	**Drying temperature(°C)**	**Target seed moisture content (%)**	**Drying time (h)**	**Drying rate (% / h)**	**Seeds temperature (^*o*^C)**
30.15 ± 1.03a*	35	9.0	36.1 ± 1.21a	0.59 ± 0.023e	32.33 ± 1.23e
30.23 ± 0.71a	40	9.0	30.3 ± 0.57b	0.70 ± 0.025 d	37.57 ± 0.88d
30.17 ± 0.94a	45	9.0	26.2 ± 0.38c	0.81 ± 0.051 c	41.63 ± 2.11c
30.28 ± 0.63a	50	9.0	22.0 ± 1.16d	0.97 ± 0.067 b	45.58 ± 1.28b
30.33 ± 0.57a	55	9.0	18.2 ± 0.71e	1.17 ± 0.041 a	49.40 ± 2.07a

IMC, initial moisture content. Values are means of four replicates. *Values followed by a different letter within a column are significantly different at the 0.05 probability level.

Thereafter, in the present study, the drying effect on the damage rate and relative electrical in sunflower seeds were further analyzed. The seed damage rate remarkably enhanced with the increase in drying temperature. This rate was found to be < 5% under temperatures of 35, 40, and 45°C. By contrast, the damage rates were 14.8 and 28.5% under temperatures of 50 and 55°C, respectively (Figures 1B,C). Consistent with the above result, the relative electrical of sunflower seeds with or without peel were significantly increased with the increase in drying temperature (Figure 1D).

### HDT Inhibited the Germination of Sunflower Seeds During Storage

**FIGURE 2:**
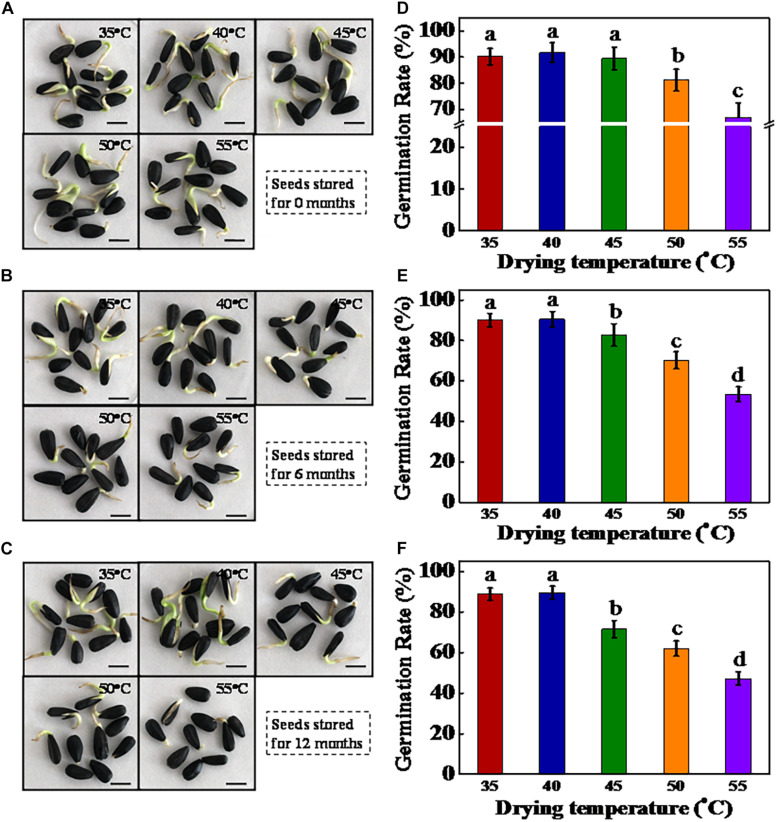
High drying temperature inhibited the germination of sunflower seeds during storage. **(A–C)** Representative photographs of sunflower seeds during the imbibition process (2 days). The dried sunflower seeds were subjected to 1, 6, or 12 months of storage before analysis. Scale bar = 10 mm. **(D–F)** Quantitative analysis on the germination rates across diverse samples **(A–C)** (on day 7 of sowing). Sunflower seeds were dried to a target moisture content of 9% at temperatures of 35, 40, 45, 50, and 55°C. Percentages represent the means from four experiments ± SE. The diverse small letter(s) on top of the bars indicate significant differences (*p* < 0.01, LSD) between treatments. Seed germination test was performed with four biological replicates.

After storage for 0, 6, and 12 months, the germinability of sunflower seeds was determined (Figure 2). HDT significantly decreased the germination rate of sunflower seeds after storage for different time periods. For sunflower seeds stored for 0 month, the germination rates of seeds dried at 50 and 55°C were significantly lower than those of seeds dried at 35, 40, and 45°C. After 6 months of storage, sunflower seeds dried at 35 and 40°C maintained a high germination rate (90%), whereas those dried at 45, 50, and 55°C showed significantly decreased germination rates (82, 70, and 53%, respectively). Moreover, the germination rate of 45°C-dried sunflower seeds was remarkably lower than those in 40°C-dried seeds after 6 months of storage, although no significant difference in germination rate was observed between the 40- and 45°C-dried seeds at 0 month of storage. A similar finding was observed in sunflower seeds stored for 12 months. Furthermore, the radicle weight and length data were also consistent with the germination results (Supplementary Figure 1). To further confirm the drying effect on sunflower seed vigor, we used AAT to obtain the artificially aged sunflower seeds. The germination rate of AAT-aged sunflower seeds significantly decreased with the increase in drying temperature (Supplementary Figure 2). In general, drying temperatures higher than 40°C reduced sunflower seed vigor during the natural and artificial aging process. The drying temperature of 40°C was evidently safe for sunflower seeds with an IMC of 30%. On clarifying the role of drying temperature (35, 40, 45, 50, and 55°C) in germination deterioration of stored sunflower seeds, only seed samples dried at 40, 45, and 50°C were used in subsequent experiments.

### HDT Induced H_2_O_2_, O_2_^–^, and MDA Over-Accumulation in Sunflower Seeds During Storage and Germination

**FIGURE 3:**
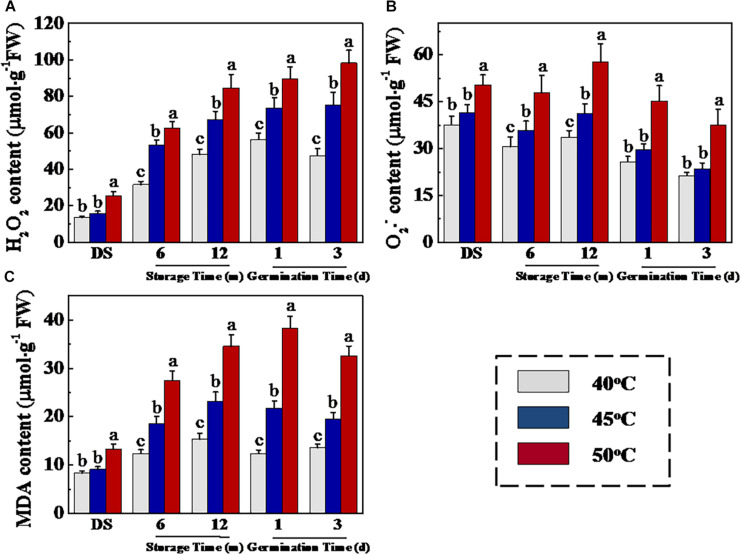
High drying temperature increased H_2_O_2_ **(A)**, O_2_^–^ **(B)**, and MDA **(C)** levels in sunflower seeds during storage and germination. Sunflower seeds were dried to a target moisture content of 9% at temperatures of 40, 45, and 50°C. The dried sunflower seeds were subjected to 1, 6, or 12 months of storage before analysis. Sunflower seeds stored for 12 months were used for germination test. Percentages represent the means from four experiments ± SE. The diverse small letter(s) on top of the bars indicate significant differences (*p* < 0.01, LSD) between treatments. The analysis of H_2_O_2_, O_2_^–^, and malondialdehyde was performed with four biological replicates.

The respiration-generated ROS contributed to seed aging. ROS over-accumulation induced lipid peroxidation by LOX, thus causing damage to cell membranes and further deteriorating seed vigor (Sharma et al., 2012; Ahmed et al., 2016). To further investigate the physiological and molecular mechanisms of drying temperature on sunflower seed deterioration, we analyzed H_2_O_2_, O_2_^–^, and MDA concentrations during storage and early germination of sunflower seeds. As is shown in Figure 3, HDT significantly elevated H_2_O_2_, O_2_^–^, and MDA levels in sunflower seeds during storage and early germination. At 0, 6, and 12 months of storage, significantly higher H_2_O_2_, O_2_^–^, and MDA levels were detected in 50°C-dried seeds compared with those in 40- and 45°C-dried sunflower seeds. Moreover, the drying temperature of 50°C significantly increased the H_2_O_2_, O_2_^–^, and MDA levels of sunflower seeds on days 1 and 3 of germination compared with temperatures of 40 and 45°C.

### HDT Increased LOX and α-Dioxygenase Activities and Transcription of Corresponding Genes in Sunflower Seeds During Storage and Germination

**FIGURE 4:**
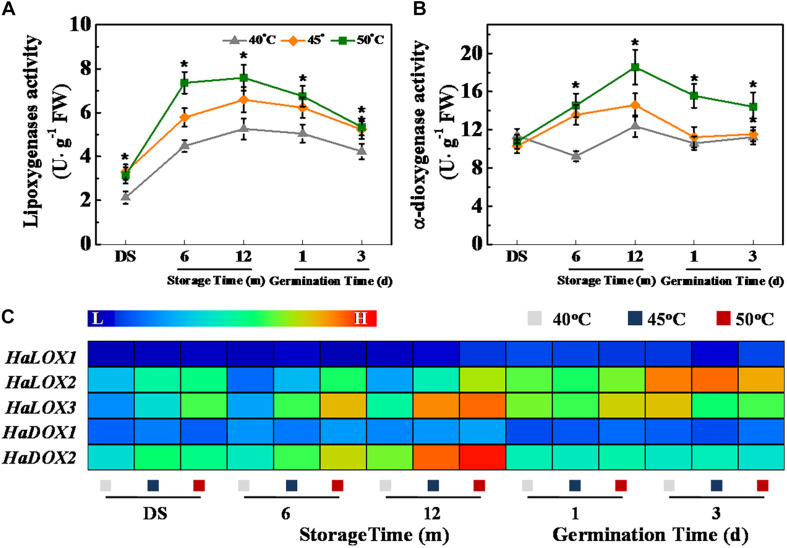
High drying temperature increased lipoxygenases **(A)** and α-dioxygenase **(B)** activities and transcription of corresponding genes **(C)** in sunflower seeds during storage and germination. Sunflower seeds were dried to a target moisture content of 9% at temperatures of 40, 45, and 50°C. The dried sunflower seeds were subjected to 1, 6, or 12 months of storage before analysis. Sunflower seeds stored for 12 months were used for germination test. LOX: lipoxygenases; DOS: α-dioxygenase. Percentages are the means of four experiments ± SE. The asterisk (*) indicates significant differences (*p* < 0.01, LSD) across treatments. The Illustrator software was used for creating the heat map. Gene expression levels from low (L) to high (H) indicated the lowest and highest levels in the entire database. The enzyme activity analysis was performed with four biological replicates. The gene expression analysis was performed with four biological replicates, and each was made in three technical replicates.

Fatty acid oxidation during storage is an important cause of toxic substance accumulation in the aging seeds, such as ROS and MDA. LOXs and dioxygenases (DOXs) represent the most frequently fatty acid oxidases (Andreou and Feussner, 2009), and we further analyzed the impact of drying temperature on the activities of these two enzymes as well as transcription of their corresponding genes (Figure 4). The LOX activity of sunflower seeds was remarkably increased with the increase of drying temperature during storage and early germination (Figure 4A). In addition, the DOS activity of 50°C-dried sunflower seeds during storage and early germination were remarkably higher than that of 40°C-dried sunflower seeds (Figure 4B). Consistently, the qPCR analysis revealed that HDT significantly up-regulated *HaLOX2*, *HaLOX3*, and *HaDX2* transcription in 6- and 12-month-stored sunflower seeds (Figure 4C).

### HDT Decreased Soluble Sugar Concentrations and ATP Level and Energy Charge in Sunflower Seeds During Storage and Imbibition Stages

**FIGURE 5:**
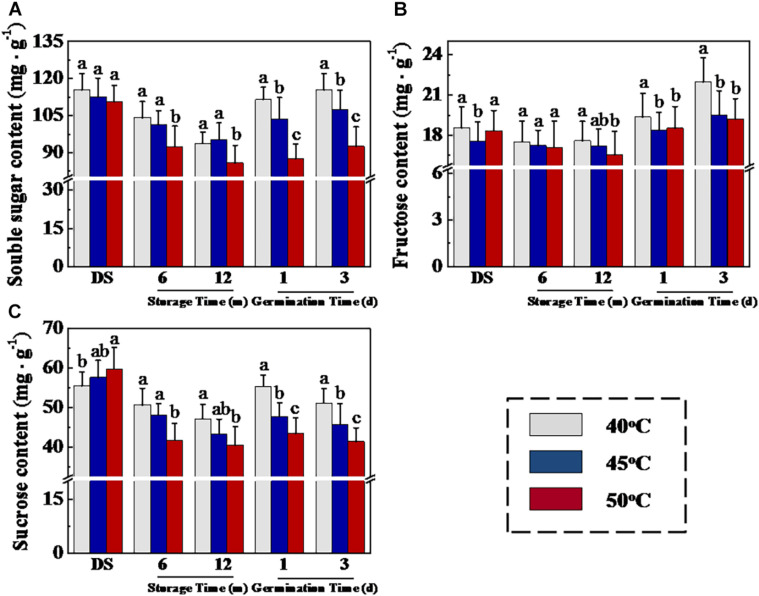
High drying temperature decreased soluble sugar **(A)**, fructose **(B)**, and sucrose **(C)** levels in sunflower seeds during storage and germination. Sunflower seeds were dried to a target moisture content of 9% at temperatures of 40, 45, and 50°C. The dried sunflower seeds were subjected to 1, 6, or 12 months of storage before analysis. Sunflower seeds stored for 12 months were used for germination test. Percentages represent the means from four experiments ± SE. The diverse small letter(s) on top of the bars indicate significant differences (*p* < 0.01, LSD) between treatments. The quantification of various sugars was performed with four biological replicates.

The seed storage substances are the major sources of energy during early seed germination and seedling emergence, and soluble sugar is the main nutrient produced by the decomposition of storage substances. To further analyze the mechanism of drying temperature in affecting sunflower seed vigor, we then determined soluble sugar, fructose, and sucrose contents in sunflower seeds (Figure 5). The results showed that HTD significantly decreased the soluble sugar, fructose, and sucrose levels in sunflower seeds at 12 months of storage. Moreover, significant higher levels of soluble sugar, fructose, and sucrose were observed in 40°C-dried sunflower seeds compared with those in 45- and 50°C-dried sunflower seeds at days 1 and 3 of germination. In addition, similar drying effects on ATP level and energy charge were shown in sunflower seeds during early germination process (Supplementary Figure 3). The above findings revealed that drying temperature affected sunflower seed germination after storage *via* the regulation of soluble sugar and ATP levels.

**FIGURE 6:**
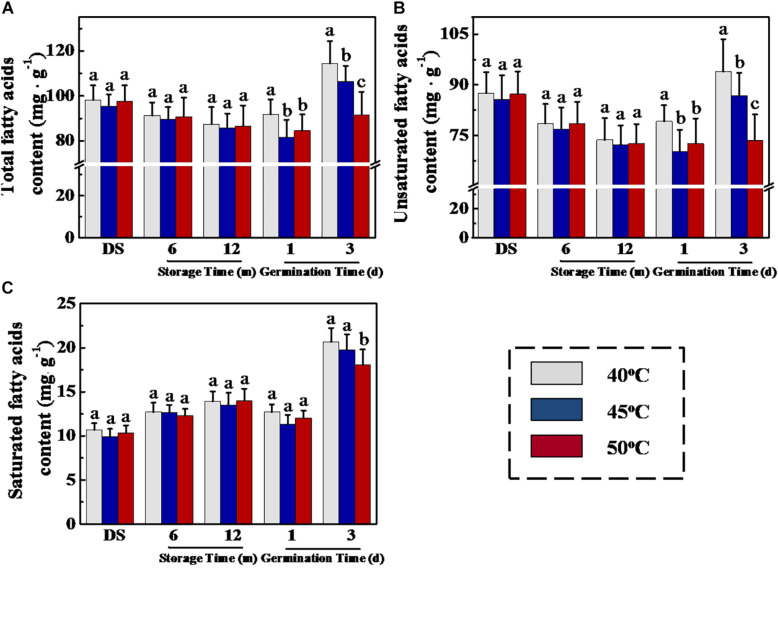
Effect of drying temperature on total fatty acid **(A)**, unsaturated fatty acid **(B)**, and saturated fatty acid **(C)** level in sunflower seeds during storage and germination. Sunflower seeds were dried to a target moisture content of 9% at temperatures of 40, 45, and 50°C. The dried sunflower seeds were subjected to 1, 6, or 12 months of storage before analysis. Sunflower seeds stored for 12 months were used for germination test. Percentages represent the means from four experiments ± SE. The diverse small letter(s) on top of the bars indicate significant differences (*p* < 0.01, LSD) between treatments. The fatty acid analysis was performed with four biological replicates.

It is well known that triacylglycerol hydrolysis in germinated oilseeds generated fatty acids and glycerol, which are subsequently transformed to different soluble sugars by gluconeogenesis (Eastmond, 2007; Quettier and Eastmond, 2009). For elucidating the reasons responsible for the effect of drying temperature on soluble sugar levels in sunflower seeds during early germination, we measured fatty acid concentration during sunflower seed storage and germination (Figure 6). In the GC-MS analysis, drying temperature demonstrated no effect on total fatty acid, unsaturated fatty acid, and saturated fatty acid concentrations in stored sunflower seeds. Whereas, on days 1 and 3 of germination, total fatty acid and unsaturated fatty acid concentrations in 40°C-dried sunflower seeds were evidently higher than those in 45- and 50°C-dried sunflower seeds. Moreover, the drying temperature of 50°C caused a significant decrease in saturated fatty acid concentration on day 3 of germination.

### Drying Temperature Regulated Several Key Enzyme Activities Involved in Fatty Acid Metabolism and Glycometabolism in Sunflower Seeds During Storage and Germination

**FIGURE 7:**
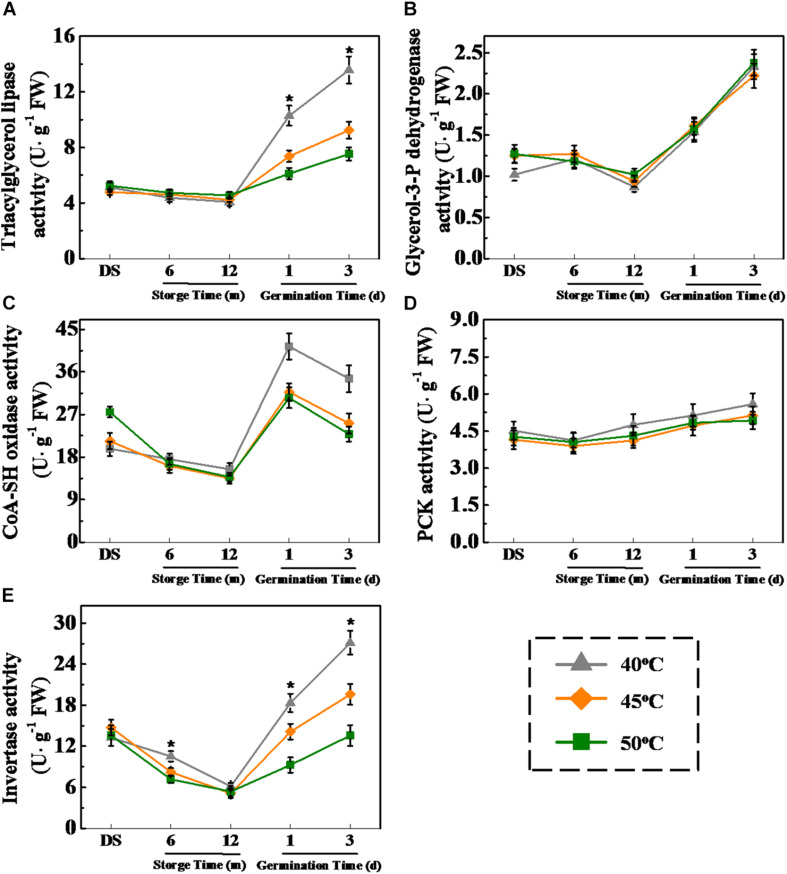
Effect of drying temperature on the activities of several key enzymes related to fatty acid metabolism and glycometabolism in sunflower seeds during storage and germination. **(A)** Triacylglycerol lipase activity; **(B)** glycerol-3-P dehydrogenase activity; **(C)** CoA-SH oxidase activity; **(D)** phosphoenolpyruvate carboxykinase (PCK) activity; and **(E)** invertase activity. Fresh sunflower seeds were dried to a target moisture content of 9% at temperatures of 40, 45, and 50°C. The dried sunflower seeds were subjected to 1, 6, or 12 months of storage before analysis. Sunflower seeds stored for 12 months were used for germination test. Percentages represent the means from four experiments ± SE. The diverse small letter(s) on top of the bars indicate significant differences (*p* < 0.01, LSD) between treatments. The enzyme activity analysis was performed with four biological replicates.

Considering that HDT decreased soluble sugar and fatty acid levels during sunflower seed imbibition, the drying effect on activities of several critical enzymes related to triacylglycerol conversion into fatty acids and sugars was examined (Figure 7). No significant difference in LIPG, ACX, and INV activities was observed in 6- and 12-month-stored sunflower seeds between different treatments. However, LIPG, ACX, and INV activities of imbibition seeds (days 1 and 3) dried at 40°C were apparently higher than that of 45- and 50°C-dried seeds. Drying temperature exerted no effect on GPDH and PCK activities during storage and early germination. These results were consistent with the drying effect on fatty acid and soluble sugar concentrations in sunflower seeds during early germination process.

### HDT Decreased the Transcription Levels of Fatty Acid Metabolism- and Glycometabolism-Related Genes in Sunflower Seeds During Storage and Germination

**FIGURE 8:**
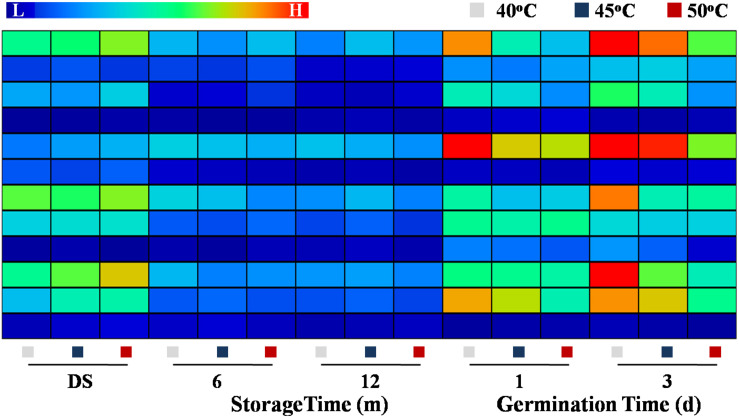
Effect of drying temperature on the transcription levels of fatty acid metabolism- and glycometabolism-related genes in sunflower seeds during storage and germination. Sunflower seeds were dried to a target moisture content of 9% at temperatures of 40, 45, and 50°C. The dried sunflower seeds were subjected to 1, 6, or 12 months of storage before analysis. Sunflower seeds stored for 12 months were used for germination test. LIPG: triacylglycerol lipase; ACX: CoA-SH oxidase; INV: invertase. The Illustrator software was used for creating the heat map. Gene expression levels from low (L) to high (H) indicated the lowest and highest levels in the entire database. The gene expression analysis was performed with four biological replicates, and each was made in three technical replicates.

The qPCR analysis revealed that the transcriptional levels of *HaLIPG1-3*, *HaACX1-3*, and *HaINV1-5* were lowly expressed during storage process and highly expressed during the early germination period. The transcription of *HaLIPG1*, *HaLIPG3*, *HaACX2*, and *HaACX4* was significantly declined with the increase of drying temperature at days 1 and 3 of germination. Similarly, the drying temperatures of 45 and 50°C significantly lowered the transcription of *HaINV3* and *HaINV4* during early germination compared with the drying temperature of 40°C (Figure 8).

*HaLIPG* encodes LIPG, a critical enzyme related to triacylglycerol hydrolysis. *HaACX* encodes ACX associated with the β-oxidation of fatty acid. *HaINV* is responsible for encoding INV, which catalyze the transformation of sucrose to fructose. As found in our study, drying temperature exerted certain effect on triacylglycerol hydrolysis and fatty acid transformation to sugars *via* regulating the key pathway-related gene expression and enzyme activities. Fructose and sucrose, the triacylglycerol hydrolysis end-products, generated ATP and energy essential to support the germination of stored sunflower seeds.

### HDT Increased ABA Level and Decreased GA Level by Affecting the Corresponding Enzyme Activities in Sunflower Seeds During Storage and Germination

**FIGURE 9:**
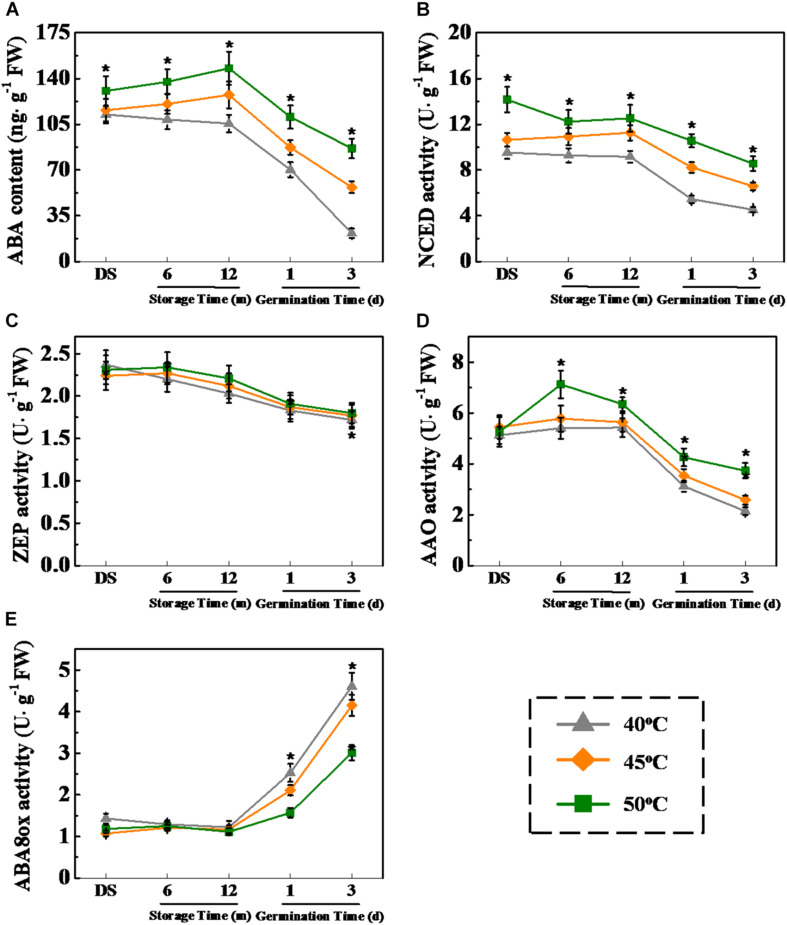
High drying temperature increased abscisic acid (ABA) level and affected corresponding enzyme activities in sunflower seeds during storage and germination. **(A)** ABA level; **(B)** 9-*cis*-epoxycarotenoid dioxygenase (NCED) activity; **(C)** Zeaxanthin epoxidase (ZEP) activity; **(D)** abscisic aldehyde oxidase (AAO) activity; and **(E)** ABA-8′-hydroxylases (ABA8ox) activity. Sunflower seeds were dried to a target moisture content of 9% at temperatures of 40, 45, and 50°C. The dried sunflower seeds were subjected to 1, 6, or 12 months of storage before analysis. Sunflower seeds stored for 12 months were used for germination test. Percentages represent the means from four experiments ± SE. The diverse small letter(s) on top of the bars indicates significant differences (*p* < 0.01, LSD) between treatments. The analysis of ABA and enzyme activity was performed with four biological replicates.

**FIGURE 10:**
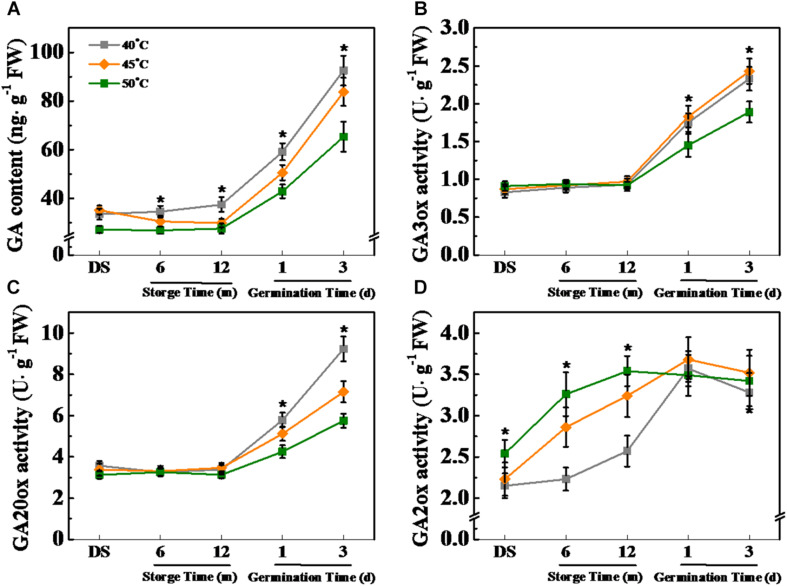
High drying temperature decreased gibberellin (GA) level and affected corresponding enzyme activities in sunflower seeds during storage and germination. **(A)** GA level; **(B)** GA3-oxidase (GA3ox) activity; **(C)** GA20-oxidase (GA20ox) activity; and **(D)** GA2-oxidase (GA2ox) activity. Sunflower seeds were dried to a target moisture content of 9% at temperatures of 40, 45, and 50°C. The dried sunflower seeds were subjected to 1, 6, or 12 months of storage before analysis. Sunflower seeds stored for 12 months were used for germination test. Percentages represent the means from four experiments ± SE. The asterisk (*) indicates significant differences (*p* < 0.01, LSD) across treatments. The analysis of GA and enzyme activity was performed with four biological replicates.

Considering the important role of GA and ABA in plant seed dormancy and germination (Shu et al., 2016, 2018), the relationship between drying effect on seed vigor and the metabolic pathways of GA and ABA during storage and early germination of sunflower seeds was investigated (Figures 9, 10). The results showed that ABA level was significantly increased with the increase of drying temperature during sunflower seed storage and early germination (Figure 9A). The drying temperature of 50°C remarkably enhanced NCED and AAO activities in sunflower seeds during storage and germination. By contrast, ABA8ox activity in 50°C-dried sunflower seeds was apparently lower than those in sunflower seeds dried at 40 and 45°C (Figure 9E).

Gibberellin level was significantly lowered by HDT (45 and 50°C) during sunflower seed storage and imbibition stages. Differently, the accumulation of GA in 40°C-dried sunflower seeds was significantly higher than that in sunflower seeds dried at 45and 50°C on days 1 and 3 of imbibitions (Figure 10A). GA3ox and GA20ox activities remained unaffected by drying temperature during sunflower seed storage and were down-regulated by HDT during early germination (Figures 10B,C). GA2ox activity was remarkably elevated with the increase of drying temperature during seed storage but remained unchanged on days 1 and 3 of imbibition (Figure 10D).

### Drying Temperature Affected the Transcription of ABA and GA Metabolism-Related Genes in Sunflower Seeds During Storage and Germination

**FIGURE 11:**
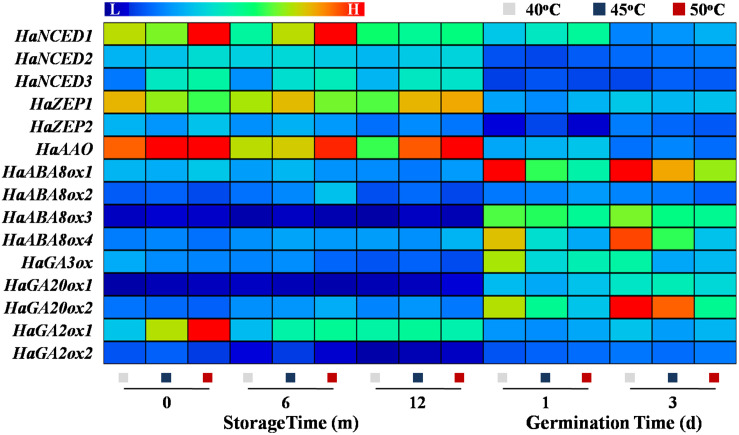
Effect of drying temperature on the transcription levels of abscisic acid (ABA) and gibberellin (GA) metabolism-related genes in sunflower seeds during storage and germination. Sunflower seeds were dried to a target moisture content of 9% at temperatures of 40, 45, and 50°C. The dried sunflower seeds were subjected to 1, 6, or 12 months of storage before analysis. Sunflower seeds stored for 12 months were used for germination test. NCED: 9-*cis*-epoxycarotenoid dioxygenase; ZEP: zeaxanthin epoxidase; AAO: abscisic aldehyde oxidase; ABA8ox: abscisic acid-8′-hydroxylase; GA3ox: GA3-oxidase; GA20ox: GA20-oxidase; GA2ox: GA2-oxidase. The Illustrator software was used for creating the heat map. Gene expression levels from low (L) to high (H) indicated the lowest and highest levels in the entire database. The gene expression analysis was performed with four biological replicates, and each was made in three technical replicates.

The qPCR analysis revealed that the expressions of ABA synthesis genes *HaNCED1*, *HaNCED3*, and *HaAAO* were remarkably increased in 50°C-dried sunflower seeds at 0 and 6 months of storage; whereas no significant effect of drying temperature on those genes expression was detected during sunflower seed early germination. The transcription levels of ABA catabolic genes *HaABA8ox1* and *HaABA8ox4* was significantly decreased with the increase of drying temperature on days 1 and 3 of imbibition but remained unchanged during storage. On days 1 and 3 of imbibition, the transcription levels of GA synthesis genes *HaGA3ox* and *HaGA20ox2* in 40°C-dried sunflower seeds was significantly elevated compared with those observed in 45- and 50°C-dried seeds. Moreover, the expression of the GA catabolic gene *HaGA2ox2* in sunflower seeds was significantly increased with the increase of drying temperature at 0 month of storage (Figure 11). The above findings revealed that drying temperature affected ABA and GA metabolism *via* the regulation of the critical enzyme activities and gene transcription levels associated with the corresponding pathways.

## Discussion

Seeds represent the foundation of agriculture, and high seed vigor is critical for crop growth and productivity (Ellis, 2011). Mechanical seed drying is among the key steps for controlling seed quality in seed production (Li et al., 2017). The present study found that HDT increased the damage rate of dried sunflower seeds and aggravated the seed deterioration during storage process, which is accompanied by the regulation of fatty acid oxidation and hydrolysis metabolism, toxic substance accumulation, and ABA/GA balance.

### Drying Temperature Exerted an Effect on Drying Performance and Sunflower Seed Characteristics

The drying rate and seed temperature are closely involved in the drying effect on crop seed vigor (Huang et al., 2020a). HDT leads to an accelerated drying rate, thereby reducing the drying time. In the present study, seed temperature and drying rate showed significant increases with the increase of drying temperature, indicating a significant positive correlation between drying temperature and seed temperature and drying rate. These findings were consistent with those reported by Hasan et al. (2014), suggesting that HDT possibly elevated drying rate by increasing the internal diffusion and transfer rates of seed water. Furthermore, drying temperature exerted a consistent effect on the drying rate and seed temperature in other crops including wheat (Ueno, 2003), table olives (Ongen et al., 2005), green chili (Hossain and Bala, 2002), strawberry (Doymaz, 2008), and tomatoes (Doymaz, 2007). Besides, HDT lead to a significant increased of damage rate and relative electrical in sunflower seeds, suggesting that HDT made a mechanical damage to the peel and seed coat of sunflower. Consistently, Igathinathane et al. (2008) reported that high-temperature drying can cause seed damage such as stress cracks and mechanical damage.

### HDT Accelerated Sunflower Seed Deterioration During Storage

The effect of mechanical drying on seed vigor has been reported in crops, such as rice (Huang et al., 2020a,b), corn (Jittanit et al., 2010), soybean (Souza et al., 2015), and wheat (Ueno, 2003). While the studies mentioned above primarily focused on the seed vigor after drying but did not monitor the dynamic process of seed vigor deterioration during storage. In the present study, it was observed that HDT of 50 and 55°C significantly reduced the sunflower seed germination rate after drying, whereas drying temperatures of 35, 40, and 45°C did not lead to any significant difference in germination rate after drying, with all seeds achieving a rate of 90%. Remarkably, seed germination rate after drying at 45°C was significantly decreased following natural aging and artificial aging process, which remained high after drying at 35 and 40°C. Therefore, it was proposed that drying temperature exerted an important effect on sunflower seed vigor. For sunflower seeds with an IMC of 30%, drying temperature higher than 40°C accelerated sunflower seed deterioration during the natural storage and artificial aging process. However, we previously reported that the upper limits of drying temperature for rice seeds increased with decreasing seed IMC (Huang et al., 2020b). It was worth noting that seed harvested with lower IMC will show less impact of HTD (Hasan et al., 2014; Souza et al., 2015).

### HDT Induced ROS and MDA Over-Accumulation in Aged Sunflower Seeds

Seed storage leads to seed deterioration, which is specifically manifested as a decrease in seed vigor. Oilseeds are rich in lipids, and lipid oxidation may be an important cause of seed degradation during storage (Kibinza et al., 2011). PUFAs, which are abundant in oilseeds, show particular susceptibility to oxidation. LOXs and dioxygenase (DOX) are the vital enzymes oxidizing PUFA (Feussner and Wasternack, 2002). The role of LOX-catalyzed lipid oxidation during seed aging has been reported in rice, which showed that the loss of LOX activity resulted in enhanced seed storage stability and reduced ROS and MDA accumulation (Gayen et al., 2014; Huang et al., 2014; Xu et al., 2015). Moreover, Devaiah et al. (2007) reported that the level of MDA, the PUFA oxidation end-product, was negatively correlated with seed vigor during natural and artificial seed aging processes in *Arabidopsis*. Excessive ROS level aggravated lipid peroxidation *via* LOX, thereby inducing cell membrane damage and accelerating seed deterioration (Sharma et al., 2012; Ahmed et al., 2016). Consistently, our study revealed that HDT significantly enhanced H_2_O_2_, O_2_^–^, and MDA levels in sunflower seeds during storage and early germination. Moreover, LOX and DOS activities and *HaLOX2*, *HaLOX3*, and *HaDOX2* transcription levels in sunflower seeds during storage was remarkably enhanced with the increase of drying temperature. It was proposed that HDT remarkably aggravated lipid oxidation, leading to ROS and MDA over-accumulation in sunflower seeds during storage. Changes in membrane lipids due to peroxidation may be involved in the increase in membrane permeability and cellular leakage that is associated with seed aging (Ma et al., 2015). HDT might increased the microfractures of sunflower pericarp and seed coat, which interrupting the impermeability to the oxygen favoring the formation of peroxide radicals in the endosperm of the seed.

### HDT Inhibited Triacylglycerol Hydrolysis to Sugars in Stored Sunflower Seeds

Soluble sugars, particularly fructose and sucrose, are the main energy sources for plant seeds during early germination (Quettier et al., 2008). In oilseeds, such as sunflower and soybean, triacylglycerol catabolism plays a vital role in providing energy for seed germination and seedling emergence. Triacylglycerol is catalyzed by LIPG to form fatty acids and glycerin; thereafter, fatty acids and glycerin are converted to sucrose *via* glycolysis, tricarboxylic acid cycle, or glyoxylate cycle, thereby providing nutrients for seed germination (Kaymak, 2014; Basnet et al., 2016). We confirmed that HDT significantly suppressed LIPG, ACX, and INV activities along with their associated gene expression in 12-month-stored sunflower seeds during the early germination stage. Consistently, total fatty acid, unsaturated fatty acid, soluble sugar, and ATP levels in HDT-dried sunflower seeds showed a significant decrease. The above results suggested that HDT suppressed the conversion of triacylglycerol and fatty acid into sucrose and decreased the sunflower seed vigor.

### HDT Affected the ABA/GA Balance in Stored Sunflower Seeds

The GA and ABA metabolism is closely associated with seed aging and germination (An and Lin, 2011; Zhang et al., 2011; Arc et al., 2013; Boccaccini et al., 2016). During storage, seeds with a low seed vigor showed high ABA level but low GA level (Lin et al., 2017). Moreover, the decrease in ABA level and increase in GA level were crucial for normal seed germination during the early imbibition process (Jia et al., 2012; Liu et al., 2016). However, whether the drying effect on sunflower seed vigor during storage and germination is associated with the ABA and GA metabolism has not yet been studied. In this study, HDT significantly increased the ABA level in sunflower seeds during storage and early germination. The transcriptions of *HaNCED1*, *HaNCED3*, and *HaAAO* increased, and the transcriptions of *HaABA8ox1* and *HaABA8ox4* decreased, thereby increasing NCED activity and lowering *ABA8ox* activity in sunflower seeds after HDT treatment. This result was consistent with the drying effect on ABA level in aged sunflower seeds. Moreover, HDT significantly increased the GA2ox activity in sunflower seeds during storage and decreased GA3ox and GA20ox activities during early germination, thereby suppressing GA accumulation in sunflower seeds. Consistently, Lin et al. (2017) reported that natural aging remarkably suppressed rice seed germination by increasing GA2ox activity and *OsGA2ox5* expression. It was proposed that GA2ox might play crucial role in the regulation of GA on seed deterioration during seed storage. The above findings suggested that HDT exerts an effect on the ABA and GA metabolism *via* changes in enzyme activities and transcription levels during seed storage and germination.

## Conclusion

**FIGURE 12:**
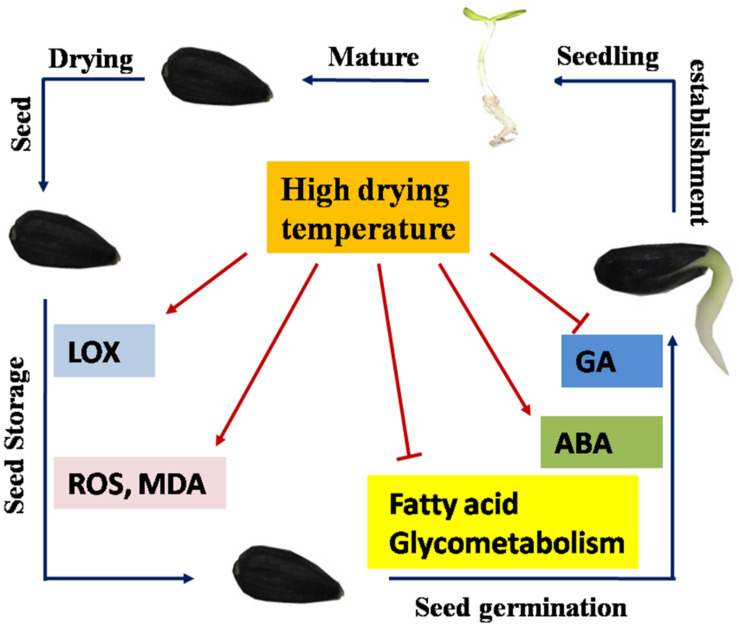
Proposed scheme for the role of drying temperature in sunflower seed vigor. Seed drying reduced the moisture of newly harvested sunflower seeds to a safe level (9%), and the dried seeds entered the storage stage. The low drying temperatures of 35 and 40°C suppressed seed deterioration during storage. However, higher drying temperatures enhanced LOX and DOX activities during seed storage and induced ROS and MDA over-accumulation. During early germination, sunflower seeds dried at a high temperature exhibited hindered conversion from triacylglycerol to fatty acid and glycerol and subsequently to soluble sugars. Moreover, a high drying temperature elevated abscisic acid (ABA) levels but reduced gibberellin (GA) levels in sunflower seeds *via* modulation of metabolism-related gene expression and enzyme activities. In conclusion, this model suggested that high drying temperature inhibited the sunflower seed vigor *via* modulation of the fatty acid oxidation and hydrolysis metabolism, toxic substance accumulation, and ABA/GA balance.

Cumulatively, the present study revealed that drying temperature exerted a substantial effect on sunflower seed vigor deterioration. A drying temperature of 40°C was evidently safe for sunflower seeds with an IMC of 30%, whereas HDT of 45, 50, and 55°C remarkably reduced sunflower seed vigor during storage and AAT process. Furthermore, HDT made a mechanical damage to the peel and seed coat of sunflower, and this may explain the significant aggravated lipid oxidation and ROS over-accumulation in sunflower seeds during storage process. During early germination, HDT remarkably suppressed triglyceride and fatty acid hydrolysis in sunflower seeds after storage. Moreover, HDT increased the ABA level and decreased the GA level by regulating enzyme activities and gene expressions in the corresponding pathways (Figure 12).

## Data Availability Statement

The original contributions presented in the study are included in the article/Supplementary Material, further inquiries can be directed to the corresponding author/s.

## Author Contributions

YH designed and performed most of the experiments. ML and HW performed the experiments of qRT-PCR analyses. TZ and PW contributed to the enzymes and HPLC analysis. DC reviewed and edited the whole manuscript. All authors read and approved the final version of the manuscript.

## Conflict of Interest

ML, HW, TZ, and PW were employed by the company Huzhou Keao Seed Co., Ltd. The remaining authors declare that the research was conducted in the absence of any commercial or financial relationships that could be construed as a potential conflict of interest.
