# Supplementary material for: High Drying Temperature Accelerates Sunflower Seed Deterioration by Regulating the Fatty Acid Metabolism, Glycometabolism, and Abscisic Acid/Gibberellin Balance
Source: Front Plant Sci. 2021 May 28;12:628251. doi: 10.3389/fpls.2021.628251 (PMC8193951; doi:10.3389/fpls.2021.628251)
Supplement: Supplementary file 1 [file Data_Sheet_1.PDF]

High drying temperature accelerates sunflower seed deterioration by regulating the fatty acid metabolism, glycometabolism, and abscisic acid/gibberellin balance

Table S1. The weather conditions during harvest time in Huzhou city, Zhejiang Province, China

Date	Air temperature (^{o}C) (maximum/minimum)	Weather conditions
October 11, 2018	20/12	Overcast
October 12, 2018	21 /11	Sunny
October 13, 2018	22 /13	Sunny

Table S2. Primers used in qRT-PCR analysis of genes expression

Gene name	Accession no.	Primer sequence	
		Forward primer	Reverse primer
Actin	XM_022142191.1	CCGCCCGAGAGGAAATATAG	GAAGGTGCTGAGTGATGCAA
HaLIPG1	XM_022132962.1	GATCCCTGAATCGTTGCCTA	TACATCTCCCAACGCATCG
HaLIPG2	XM_022140162.1	CAAATATAGTCGCGGTCATGC	ACTCATCCCATGTCCAATCC
HaLIPG3	XM_022115698.1	AGCACGAGAGAATTCAACAGG	GTAGTGGAGGGTGGCGTCT
HaACX1	XM_022160757.1	TTCACAAGCATCAAGGTGATTT	GTGAGGCCTGTTTGGGAGTA
HaACX2	XM_022182799.1	AAGTTGATGCCAATGTTAGCC	TCAATCAAATGCACGGTTG
HaACX3	XM_022167322.1	TGTGACTTATCAAACTGCTGAGG	TGTAGAATCAAACGATCCGAGA
HaACX4	XM_022137461.1	TGGCCTTAGAACAACAGCAA	CATCGTTTTTGGCCTTCAAT
HaINV1	XM_022122741.1	TCCTAAGAACACTCCTTGGTCAT	AACTGCCAAAGAAGCGTAGG
HaINV2	XM_022164742.1	TCAACGGCCATTATGGATCT	AACCTTAACAGGCATATCTCCAA
HaINV3	XM_022179071.1	TCGGCGATTATGGATCTTTT	TCTTAATAGGCATTTCACCAACG
HaINV4	XM_022142015.1	ATGTGTGCACGTGAAATGCT	AAGACGGTTGTTTAACGCTCTAAT

HaINV5	XM_022164892.1	GCGCAAACAACGGCTATTAT	ATCTCGCCAATCAGGTCTTC
HaNCED1	XM_022179446.1	TGATGAAAAGACATGGAAATCG	GAAGCTTCCATCTTCATTGTAACC
HaNCED2	XM_022157417.1	ATTTCACCGGAAAATGACGTT	GCCGAGGAGGTGTTTGTTC
HaNCED3	XM_022171044.1	CGTCACGTTGGGACCTAATAAT	ACCGAGCGATAATTCTTGTTG
HaZEP1	XM_022179683.1	AAGGAAAGAAGGGTTCGGATA	AGCTAGCACCAGACCACCAA
HaZEP2	XM_022142950.1	ATCTTTGGAGGCTGGTGTGA	ATTGCTTCTTCATCGGTTGC
HaAAO	XM_022170388.1	CGTTCGATAGAGGAACTCACG	CCATTACACCGTTGTCAGCA
HaABA8ox1	XM_022114231.1	TGAAGATGTTGAATTTGAAGGGTA	TGAATGTTTCTAAAGAGTGGCAAT
HaABA8ox2	XM_022181633.1	GATCTCATACGGCGGAGCTA	CATTGAGCCTGGCGGTAG
HaABA8ox3	XM_022136531.1	TGCATCATTGCCCTTGAATA	TATCCCGAGCCTTCATACCA
HaABA8ox4	XM_022183023.1	CACAATCCCGAATTCTTTCC	TGGCACCTCGAATCTTGAC
HaGA3ox	XM_022154428.1	GACGATAGCCCAACCTCGT	GCATCATGGCATTAGGGTCT
HaGA20ox1	XM_022178611.1	TTTGATGCATCGGTCCTACA	GATCCGGCCATATGAACTGT
HaGA20ox2	XM_022158755.1	TTCACGGCATTATCAAATGG	TGGGGACAAACTGTTCACAC
HaGA2ox1	XM_022157397.1	TTCGGTTACGGGAACAAAAG	AAGAAGGTATTCGACCCAACC
HaGA2ox2	XM_022122742.1	ACACTTGGCTCCCTGTTGAC	GCCTGCAAGAGATCACCAAT
HaLOX1	XM_022124717.1	AAGAACCTCGGGTTTCCATAC	GGCAGATTAACACCCTCATTG
HaLOX2	XM_022170774.1	TTGGTCACGGAGACAAGAAA	AATCGTCTTGGGTTTTGAGC
HaLOX3	XM_022170775.1	TACCTGCGGACCTAATCAGC	GCTAGCTTTAAACCATGTGGAGA
HaDOX1	XM_022149844.2	AGCATCACTCGACATTTATAGGG	TCTACGGAACTCATTGTATCTTGC
HaDOX2	XM_022173842.2	CAGCAGGTTGAGATTGTAGCTC	AAAACTTAAATGACTTCAATGGAC

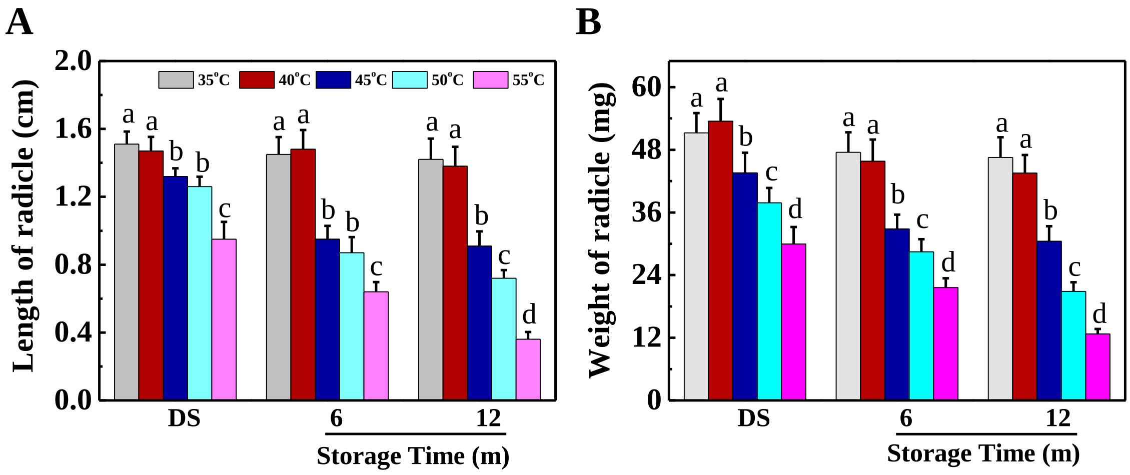

Fig.S1. High drying temperature inhibited the germination of sunflower seeds during storage. (A) The radical length of germinated sunflower seeds was measured (2 days after sowing). (B) The radical weight of germinated sunflower seeds were quantified (2 days after sowing). Sunflower seeds were dried to a target moisture content of 9% at temperatures of 35 ℃, 40 ℃, 45 ℃, 50 ℃, and 55 ℃. Sunflower seeds were stored for 0, 6, or 12 months after drying and then subjected to analysis. Percentages represent the means from four experiments ±SE. The diverse small letter(s) on top of the bars indicate significant differences ($p<0.01$, LSD) between treatments.

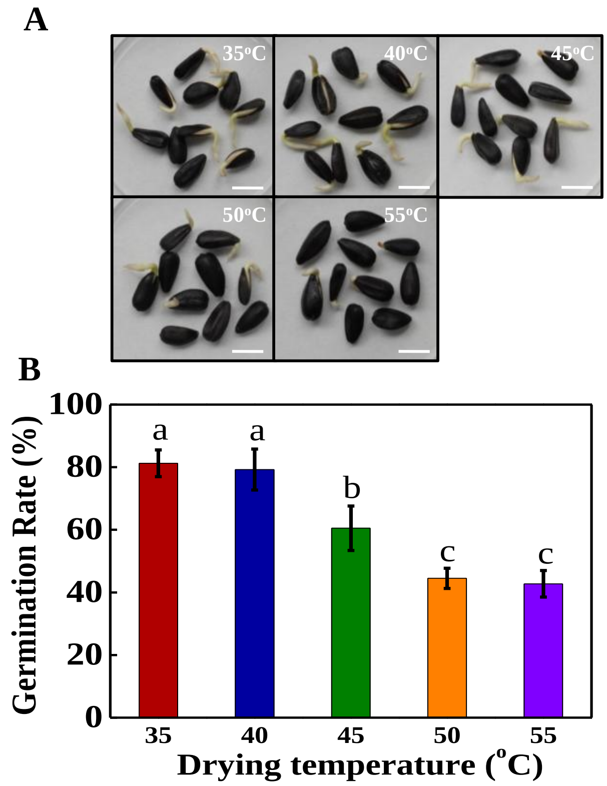

Fig.S2. High drying temperature inhibited the germination of AAT-aged sunflower seeds. (A) Representative photographs of sunflower seeds aged using the accelerated aging test (AAT) during the imbibition process (2 days). Scale bar=10 mm. (B) The quantitative analysis of final germination rates of (A) are shown (7 days after sowing). Sunflower seeds were dried to a target moisture content of 9% at temperatures of 35 ℃, 40 ℃, 45 ℃, 50 ℃, and 55 ℃. Percentages represent the means from four experiments ±SE. The diverse small letter(s)

on top of the bars indicate significant differences (p<0.01, LSD) between treatments.

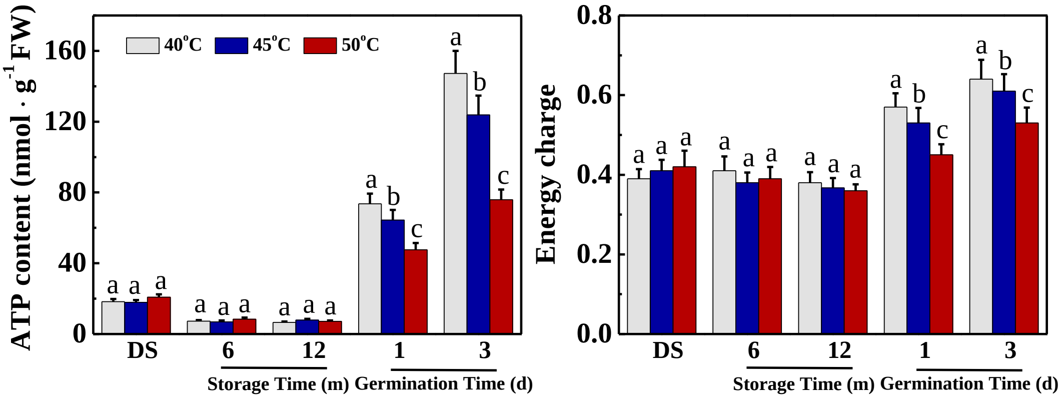

Fig.S3. High drying temperature decreased ATP content (A) and energy charge (B) in sunflower seeds during germination. Sunflower seeds were dried to a target moisture content of 9% at temperatures of 40 ℃, 45 ℃, and 50 ℃. The dried sunflower seeds were subjected to 0, 6, or 12 months of storage before analysis. Sunflower seeds stored for 12 months were used for germination test. Percentages represent the means from four experiments ±SE. The diverse small letter(s) on top of the bars indicate significant differences (p<0.01, LSD) between treatments.
